# Clinical impact of collagen sponge combined with substance P on maxillofacial trauma repair in rats

**DOI:** 10.3389/fmed.2025.1631988

**Published:** 2025-10-16

**Authors:** Qian Wang, Yong Zhang, Yan Guo, Xiaolan Yang

**Affiliations:** ^1^School of Stomatology, Quanzhou Medical College, Quanzhou, Fujian, China; ^2^Department of Burn Intensive Care Unit, Quanzhou First Hospital, Quanzhou, Fujian, China

**Keywords:** maxillofacial trauma, collagen sponge, substance P, trauma healing, TGF-β1/Smad3-VEGF signaling pathway

## Abstract

**Objective:**

Collagen sponge and substance P (SP) can facilitate trauma healing. Therefore, this study aimed to clarify the role and mechanism of collagen sponge combined with SP in maxillofacial trauma healing.

**Methods:**

A maxillofacial trauma model was established using rats. The rats were divided into four groups: research group 1 (RG 1; Vaseline gauze and SP), research group 2 (RG 2; Vaseline gauze and collagen sponge), research group 3 (RG 3; collagen sponge and SP), and a control group (CG; Vaseline gauze and normal saline). The trauma healing rate was examined on days 3, 7, and 14 after treatment, and the pathological morphology of the trauma was examined on day 14 after treatment. Hydroxyproline expression; positive expression of basic fibroblast growth factor (bFGF); serum IL-6 and TNF-*α* levels; matrix metalloprotease-9 (MMP-9) and tissue inhibitor of metalloproteinase-1 (TIMP-1) protein levels; and vascular endothelial growth factor (VEGF), TGF-β1, and SMAD3 mRNA levels were detected on day 14 after treatment.

**Results:**

On days 7 and 14 after treatment, the trauma healing rates in all research groups were higher than that in the CG, and the trauma healing rate in RG 3 was higher than that in RG 1 and RG 2. On day 14 after treatment, compared to the CG, the number of inflammatory cells in the rat trauma tissues was reduced in all research groups, while the collagen fiber content was enhanced in all research groups. Compared to RG 1 and RG 2, the number of inflammatory cells in RG 3 was lower, while the collagen fiber content in RG 3 was higher. The tissue hydroxyproline and bFGF levels in all research groups were higher than those in the CG, and the levels in RG 3 were higher than those in RG 1 and RG 2. Compared to the CG, serum levels of IL-6 and TNF-*α* and tissue levels of MMP-9 were lower across all research groups, while tissue levels of TIMP-1 were elevated in all research groups. The improvements in serum IL-6 and TNF-*α* levels and tissue MMP-9 and TIMP-1 levels in RG 3 were greater than those in RG 1 and RG 2. Compared to the CG, the mRNA levels of VEGF, TGF-β1, and SMAD3 were higher in all research groups, with RG 3 showing higher levels than RG 1 and RG 2.

**Conclusion:**

Collagen sponge and SP can facilitate the healing of maxillofacial trauma and reduce scar formation after trauma healing. This effect may be related to the TGF-β1/Smad3-VEGF signaling pathway.

## Introduction

As the largest organ in the human body and the first line of defense, the skin is most vulnerable to external invasion. Due to their relatively exposed state, the maxillofacial regions are more susceptible to skin trauma. The maxillofacial region plays a critical role in human esthetics, and any damage to this region has a marked impact on appearance and function (1, 2). Therefore, the treatment of maxillofacial trauma must consider the principles of plastic surgery and minimize scar formation after trauma healing. Traditional dressings (such as petrolatum and silver ion gauze) can reduce infection, but they do not significantly accelerate healing. In contrast, therapies that utilize growth factors such as epidermal growth factor/fibroblast growth factor (EGF/FGF) or single biomaterials (collagen scaffold) have limited effectiveness in treating complex wounds (3, 4). This limitation is largely due to their lack of dynamic regulation capabilities and the need for frequent administration,

Collagen sponge is made from cow tendons. After selecting and removing impurities, fat, and fascial tissue, the tendons are cleaned and disinfected; purified type I collagen is prepared through enzymatic hydrolysis and acid, alkali, and organic solvent treatments, followed by cross-linking, freezing, and drying to form a sponge-like structure (5). Collagen sponge has the following functions (6, 7): It can be degraded, with a certain degree of hydroscopicity, expanding upon contact with blood; it can induce the derivation and aggregation of fibroblasts, stimulate the production and arrangement of collagen, promote the formation of capillaries, ensure blood and oxygen supply required for tissue formation, and facilitate trauma healing; it can induce mineral deposition, facilitate the formation of new bones, and induce osteogenesis; it can induce chemotactic monocytes, macrophages, and white blood cells to enhance local resistance; and it has anti-fibrinolytic properties and remarkably enhances the hemostasis process. Currently, collagen sponge is widely used in clinical practice for trauma repair, tissue filling, and other applications, achieving good therapeutic effects (8). However, when used alone, a collagen sponge may cause delayed angiogenesis due to the lack of bioactive molecules.

Substance P (SP), the first discovered brain–gut peptide substance, is widely distributed in the central nervous system and peripheral nervous system. Previous research has demonstrated a close association between SP and trauma healing (9, 10). Trauma healing is a complex biological process that involves inflammation, granulation tissue formation, matrix deposition, such as collagen fibers, and reconstruction of the epidermis, which is a complete set of biological self-repair processes. SP plays a role in the process of trauma healing, and growth factors involved in trauma repair include vascular endothelial growth factor (VEGF), epidermal growth factor (EGF), and basic fibroblast growth factor (bFGF) (11). Therefore, SP may affect growth factors. SP can directly facilitate DNA synthesis and cell proliferation in endothelial cells and fibroblasts (12), and it can also facilitate neovascularization (13). Moreover, it has been depicted that the positive rate of SP in hypertrophic scar fibroblasts is markedly higher than that in normal skin fibroblasts (14), indicating that SP may play a role in repressing scar tissue proliferation.

In view of this, this study prepared maxillofacial skin and soft tissue trauma models and clarified the role and mechanism of collagen sponge in combination with SP in maxillofacial trauma repair, which may provide a scientific basis for human skin repair. This study is the first to combine collagen sponge with SP using covalent cross-linking technology, thereby creating an intelligent dressing that offers both dynamic signal regulation and structural support functions.

## Materials and methods

### Animals

A total of 45 healthy adult Wistar rats without specific pathogen grading, both male and female and weighing 160–210 grams, were purchased from Vital River (Beijing, China). Before the experiments, all rats were fasted for 12 h and were not restricted from drinking water. All experimental protocols were approved by the Ethics Committee of Quanzhou First Hospital.

### Sample size calculation

Power analysis was carried out in this study using the G*Power 3.1.9.7 software to determine the sample size required to detect statistical differences. With an alpha level of 0.05 and 90% power analysis, the study determined that a sample size of 15 rats per group was required.

### Rat models of maxillofacial trauma

The Wistar rats were subjected to peritoneal anesthesia with 3% pentobarbital sodium (150 mg/kg, P3761, Sigma-Aldrich, United States) to create full-thickness skin trauma in the maxillofacial region of the rats. In other words, a surgical knife was used to make random 0.5 cm × 0.5 cm full-thickness skin incisions at three locations on the maxillofacial region of the rats. The wounds were treated with normal saline gauze.

### Debridement of rat maxillofacial trauma

The trauma wounds were thoroughly cleaned using physical methods. After bleeding cessation, the trauma wounds were repeatedly rinsed with normal saline and diluted iodine. After cleaning the wounds, they were covered with normal saline gauze for further treatment.

### Grouping

This study used a self-controlled design. The rats were randomly divided into four groups of 15 each: a control group (CG), research group 1 (RG 1), research group 2 (RG 2), and research group 3 (RG 3). In the CG, maxillofacial trauma was treated with Vaseline gauze (Anshi Medical Group Limited, China) of the same size as the wound, and normal saline was sprayed every 6 h. In RG 1, maxillofacial trauma was treated with Vaseline gauze of the same size as the wound and sprayed with SP (10^−7^ M, HY-P0201, MedChemExpress, United States) every 6 h. In RG 2, maxillofacial trauma was filled with a collagen sponge (Guangzhou Tratuer Biotechnology Co., LTD, China) of the same size as the wound and treated with Vaseline gauze of the same size as the wound and was every 6 h. In RG 2, maxillofacial trauma was filled with a collagen sponge (Guangzhou Tratuer Biotechnology Co., LTD, China) of the same size as the wound, covered with Vaseline gauze of the same size, and sprayed with normal saline every 6 h. The samples were collected 14 days after treatment. The experimental work of this research was conducted by three researchers.

### Observation of rat maxillofacial trauma healing

The trauma healing rates of the rats in all groups on days 3, 7, and 14 after treatment were compared. The trauma healing rate refers to the percentage of the postoperative trauma contraction area to the original trauma area. The healing status of the wound and the contraction of the wound area were observed and photographed. The electronic images were saved to a computer, and the wound area in the images was measured using the Image J software (from the National Institutes of Health, United States) according to methods described in other studies (15). Trauma healing rate = (original trauma area - actual measured area) / original trauma area × 100%.

### Hematoxylin and eosin staining

Samples were collected from the rats in the three research groups 14 days after treatment. After fixation, the tissue specimens underwent routine dehydration and were soaked in wax. The tissue specimens were then sectioned into 4-um-thick continuous slices and mounted on glass slides coated with polylysine. After staining with hematoxylin and eosin (Co105M, Beyotime, China), the histopathological structure of the tissue was observed using optical microscopy. The thickness of the newly formed epithelium was measured once per field of view under optical microscopy (40 ×, Leica, Germany), and the average value across five fields of view was calculated. An investigator blinded to the treatment condition assessed the extent of inflammation: grade 0, no inflammation; grade 1, inflammatory infiltration covering up to 5% of the skin wound tissue; grade 2, 6–10%; grade 3, 11–30%; grade 4, 31–50%; and grade 5, 51–70%.

### Masson’s trichrome staining

The collected skin wound tissue was fixed in a 4% paraformaldehyde solution, dehydrated through a graded ethanol series, and embedded in paraffin to prepare 4 μm sections. The sections were then preheated and stained with the mixture according to the instruction manual (Servicebio, China), followed by 1% glacial acetic acid differentiation for several seconds. After dehydration, the tissue sections were sealed and evaluated for collagen maturation using Masson’s trichrome staining. Quantitative analysis of Masson’s trichrome staining was performed using the ImageJ software. The quantitative analysis of collagen staining (also known as the volume fraction of collagen) was calculated as the ratio of the positive collagen area to the total tissue area.

### Detection of hydroxyproline expression

On day 14 after treatment, the skin tissue specimens were rinsed with normal cold saline, dried with filter paper, and weighed. The tissue was cut into small pieces with ophthalmic scissors, placed in a glass homogenizer tube, and ground with normal saline to prepare a 10% skin tissue homogenate, followed by centrifugation at 3000 r/min at 4 °C for 10 min. The hydroxyproline content was measured using hydroxyproline kits based on the alkaline hydrolysis method.

### Detection of bFGF-positive expression

On day 14 after treatment, immunohistochemical staining of the tissue sections was performed according to the SABC method, following the instructions provided with the kits. The anti-bFGF antibody (1/200, BS-0217R, Thermo Fisher, United States) was diluted, and the primary antibody was replaced with PBS as a negative control, followed by observation under a microscope after sealing. The appearance of brownish-yellow or brownish particles in the cytoplasm (membrane) was considered positive. A total of 10 random high-power fields were selected, the number of positive cells in each field was observed, and the positive expression rate was calculated.

### Detection of interleukin-6 and tumor necrosis factor alpha expression

On day 14 after treatment, 0.5 mL of venous blood was collected on an empty stomach. After anticoagulation, the blood specimens were centrifuged for 15 min at 3000 r/min. After standing for 10 min, the upper serum was collected and stored at −20 °C. Serum IL-6 and TNF-*α* levels (pg/mL) were detected using enzyme-linked immunosorbent assay (ELISA, ml064292 and ml002859, Shanghai Enzyme Linked Biotechnology Co., LTD, China).

### Detection of matrix metalloprotease-9 and tissue inhibitor of metalloproteinase-1 protein expression

Skin tissue specimens were obtained, and total protein was extracted using RIPA lysis buffer (Beyotime, China) and quantified using a bicinchoninic acid protein assay kit (Thermo Fisher Scientific, United States). Subsequently, 30 μg of the protein sample was separated by 10% SDS-PAGE and transferred onto PVDF membranes. The membranes were blocked in 5% non-fat milk for 2 h at room temperature and incubated with primary antibodies, including MMP-9 (1/1000, ab76003, Abcam, UK), TIMP-1 (1/1000, ab211926, Abcam, UK), bFGF (1/1000, ab222932, Abcam, UK), and *β*-actin (1/1000, ab5694, Abcam, UK). β-actin served as the internal reference control. The membranes were then incubated with a horseradish peroxidase-labeled secondary antibody (1/2000, ab6721, Abcam, United Kingdom) for 1 h at room temperature. Protein bands were visualized using an ECL kit (Amersham, UK, Product No. BB-3501), and images were captured using a Bio-Rad Image Analysis System (Bio-Rad, Hercules, CA, United States). The ImageJ software was used to measure the optical density of the protein bands. Each Western blot assay was performed in triplicate.

### Detection of TGF-β1, SMAD3, and VEGF expression

Briefly, total RNA was extracted from the skin tissue specimens using an RNA Extraction Kit (Promega, United States) according to the manufacturer’s instructions. To obtain a template for qRT-PCR, cDNA was synthesized using a PrimeScript RT Master Mix (Takara, Japan) following the manufacturer’s directions. The Power SYBR Green kit (Invitrogen, United States) was employed to perform qRT-PCR on an ABI 7900HT instrument (Applied Biosystems, United States). All PCR assays were performed in triplicate for each sample, and the 2^−ΔΔCt^ method was used for relative quantification. GAPDH was utilized as an internal control. The primer sequences used for qRT-PCR were as follows: TGF-β1: Forward, 5’-GACCGCAACAACGCAATCTA-3′, Reverse, 5′- ACTGCTTCCCGAATGTCTGA-3′; SMAD3: Forward, 5’-CATGGGCAAATGAAAGGGCT-3′, Reverse, 5’-CCAGGGTGAAGATGACAGGT-3′; VEGF: Forward, 5’-GGAACTAGACCTCTCACCGG-3′, Reverse, 5’-CTCTCCCTTCATGTCAGGCT-3′; and GAPDH: Forward, 5′- TGCTGAGTATGTCGTGGAGTCT-3′, Reverse, 5’-CAGTCTTCTGAGTGGCAGTGAT-3′.

### Statistical analysis

The SPSS 27.0 statistical software was used to perform statistical analysis. Measurement data were expressed as mean ± standard deviation (x ± s). If the data followed a normal distribution, one-way analysis of variance (ANOVA) was used for comparison among the different groups; otherwise, the Kruskal–Wallis test was employed. For time-related measurements, repeated measures analysis of variance (for parametric data) or the Friedman test (for non-parametric data) was applied to assess the within-group changes over time. If significant differences were found, appropriate *post hoc* tests (such as Tukey’s HSD test for ANOVA and Dunn’s test for the Kruskal–Wallis test) were conducted to determine which groups had significant differences. A *p*-value of < 0.05 indicated a significant difference.

## Results and discussion

Most maxillofacial injuries are caused by various factors, such as trauma, infection, and tumor-related surgery. If the trauma is not repaired in time or is improperly treated, it often leads to scar healing, which seriously affects appearance and function, thereby causing physical and mental harm to patients and leaving psychological shadows. Therefore, achieving scar-free trauma healing has always been an urgent issue in oral cosmetic surgery.

SP is widely distributed in the central and peripheral nervous systems, as well as in tissue organs. Its major physical function is to sense and defend against various harmful stimuli and maintain and repair the structure and function of damaged regions. SP can markedly accelerate the healing process of trauma, induce the concentration of epidermal stem cells at the trauma edges, and promote their migration into the granulation tissue. SP can also stimulate microvascular endothelial cells to produce growth factors during skin injury repair (16). Research has shown that after skin injury in fetal rabbits, the positive expression of SP significantly decreases, suggesting that neuropeptide SP downregulation may play a crucial role in scar-free healing of fetal rabbit skin (17). SP can induce the expression of growth factors in granulation tissue fibroblasts *in vitro*, thereby facilitating fibroblast proliferation, accelerating granulation tissue formation, and enhancing trauma healing. Collagen sponge is a biomedical material with a structure similar to that of human collagen (18). It, with a mesh-like porous structure, can induce repair cell infiltration and proliferation in surgical dressings (19). The collagen content is as high as 98%, which stops bleeding, enhances trauma healing, and enables sustained release in small doses (20). Collagen sponge also possesses excellent biocompatibility, biodegradability, and low antigenicity and can be degraded and absorbed by collagenase during the healing process. After covering the trauma, the collagen sponge can be degraded by the body, ultimately producing various amino acids that can be absorbed by the body and provide nutrients for trauma repair (21). Collagen sponge and SP may be complementary to each other. Collagen sponge provides a three-dimensional scaffold, guiding SP to act on the base and deep layers of the wound, avoiding the problem of insufficient local concentration caused by drug diffusion. The degradation cycle of a collagen sponge perfectly matches the repair-promoting window period of SP, ensuring that SP continuously exerts its effects during critical stages. The combined application of collagen sponge and SP has the following advantages: it overcomes the limitations of a single intervention measure, enhances the therapeutic effect, and reduces complications; it also meets the diverse clinical demands for “rapid healing-functional recovery-aesthetic improvement” [sic] and provides a new strategy for the treatment of complex wounds.

**Figure 1:**
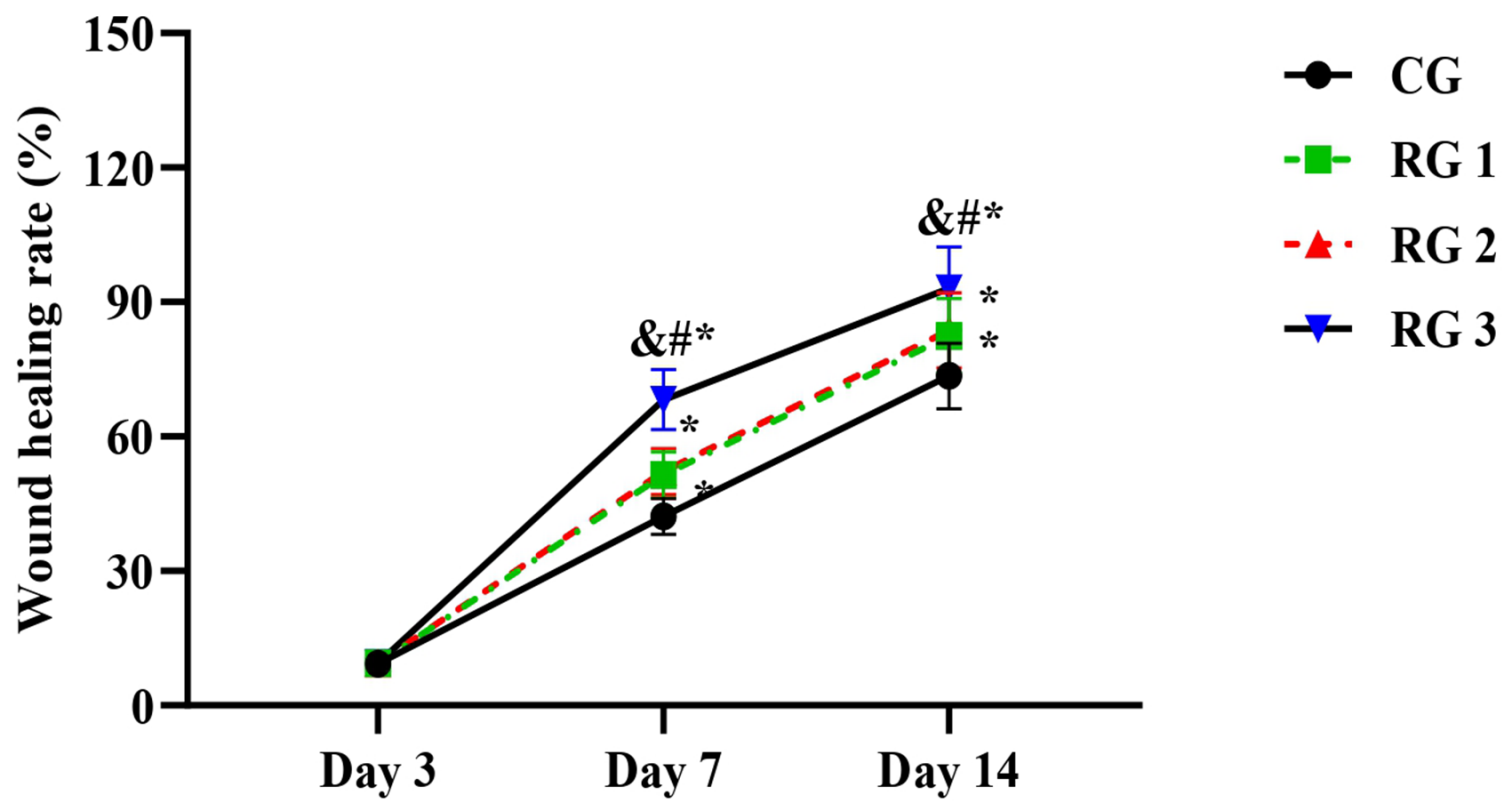
Trauma healing rate in the four groups. Versus the CG, *p < 0.05; versus RG 1, #*p* < 0.05; versus RG 2, &*p* < 0.05.

**Figure 2:**
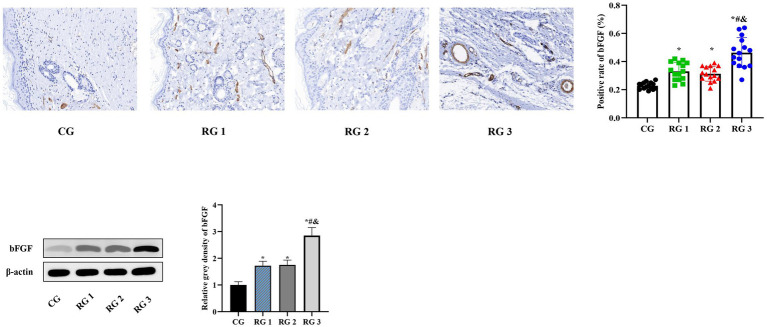
H&E and Masson’s trichrome staining of the rat trauma tissue in the four groups. Versus the CG, *p < 0.05; versus RG 1, #*p* < 0.05; versus RG 2, &*p* < 0.05.

In this study, the results indicated that 3 days after treatment, no statistically significant difference in the trauma healing rate was observed among the four groups (*p* > 0.05). On days 7 and 14 after treatment, trauma healing rates in all research groups were higher than those in the CG (*p* < 0.05); the trauma healing rate in RG 3 was higher than that in RG 1 and RG 2 (*p* < 0.05; Figure 1; Table 1). In addition, on day 14 after treatment, many inflammatory cells gathered and infiltrated the trauma sites in the CG, and a small number of fibers and collagen were deposited, forming granulation tissue. Compared to the CG, the number of inflammatory cells in the rat trauma tissue of all research groups was reduced, and local red blood cells were observed to overflow outside the blood vessel wall, accompanied by substantial collagen deposition and increased granulation tissue. Compared to RG 1 and RG 2, the number of inflammatory cells in the rat trauma tissue of RG 3 was lower, collagen fibers were clearly visible and arranged in an orderly manner, and granulation tissue at the wound site showed significant growth (Figure 2; Tables 2, 3). These findings indicate that the combination of collagen sponge and SP can accelerate the healing of maxillofacial trauma in rats, which is consistent with previous reports (22).

**Table 1:** Trauma healing rate in the four groups (%).

Groups	*n*	Day 3	Day 7	Day 4
CG	15	9.35 ± 0.93	42.17 ± 4.02	73.52 ± 7.35
RG 1	15	9.39 ± 0.96	51.45 ± 5.13^*^	82.48 ± 8.29^*^
RG 2	15	9.38 ± 0.95	52.19 ± 5.16^*^	83.65 ± 8.38^*^
RG 3	15	9.44 ± 0.95	68.22 ± 6.71^&#*^	93.03 ± 9.23^&#*^

Versus the CG, *p < 0.05; versus RG 1, #*p* < 0.05; versus RG 2, &*p* < 0.05.

**Table 2:** Histopathological score (inflammation) in the four groups (points).

Groups	*n*	Histopathological score
CG	15	4.23 ± 0.39
RG 1	15	2.75 ± 0.29^*^
RG 2	15	2.76 ± 0.28^*^
RG 3	15	1.82 ± 0.15^&#*^

Versus the CG, *p < 0.05; versus RG 1, #*p* < 0.05; versus RG 2, &*p* < 0.05.

**Table 3:** Collagen fiber content rate in the four groups (%).

Groups	*n*	Collagen fiber content rate
CG	15	17.55 ± 1.85
RG 1	15	25.55 ± 2.45^*^
RG 2	15	25.34 ± 2.64^*^
RG 3	15	42.05 ± 4.25^&#*^

Versus the CG, *p < 0.05; versus RG 1, #*p* < 0.05; versus RG 2, &*p* < 0.05.

**Figure 3:**
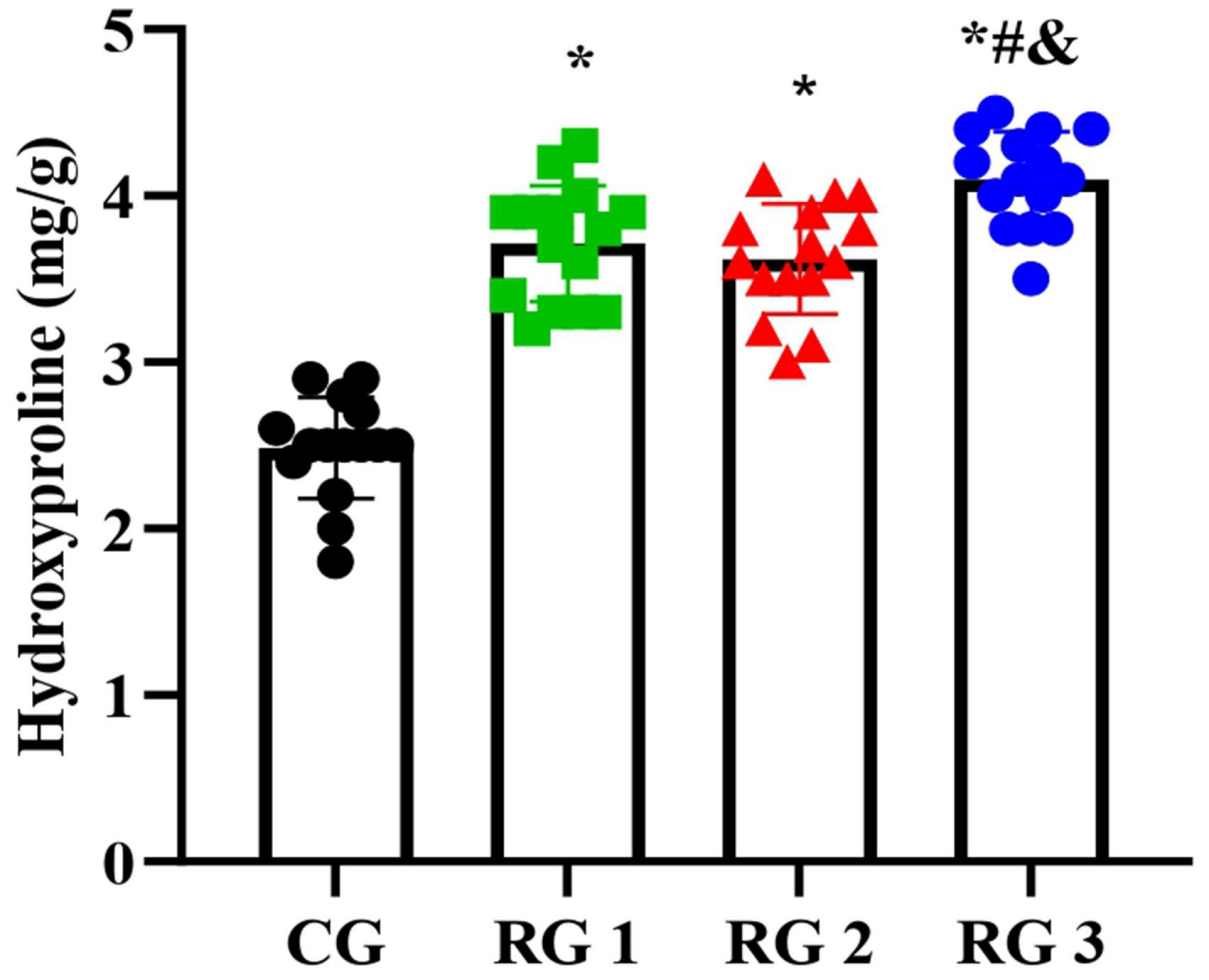
Hydroxyproline levels in the four groups. Versus the CG, *p < 0.05; versus RG 1, #*p* < 0.05; versus RG 2, &*p* < 0.05.

Hydroxyproline is a non-essential amino acid and a major, relatively constant component of collagen fibers and proteins. When collagen metabolism changes, the hydroxyproline content also changes accordingly (23). Therefore, the hydroxyproline content in skin tissue can indirectly reflect the condition of trauma healing. The results of this study indicated that on day 14 of treatment, tissue hydroxyproline contents in all research groups were elevated compared to those in the CG (*p* < 0.05). The tissue hydroxyproline content in RG 3 was elevated compared to that in RG 1 and RG 2 (*p* < 0.05; Figure 3; Table 4). Under collagen sponge intervention, the hydroxyproline content in the rat skin tissue presented a remarkable increase, indicating that the combination of collagen sponge and SP can facilitate the production and secretion of hydroxyproline in the skin, thereby elevating the synthesis and precipitation of collagen, which is beneficial for tissue regeneration and recovery.

**Table 4:** Hydroxyproline content in the four groups (mg/g).

Groups	*n*	Hydroxyproline
CG	15	2.48 ± 0.30
RG 1	15	3.71 ± 0.34^*^
RG 2	15	3.62 ± 0.33^*^
RG 3	15	4.10 ± 0.28^&#*^

Versus the CG, *p < 0.05; versus RG 1, #*p* < 0.05; versus RG 2, &*p* < 0.05.

Basic fibroblast growth factor (bFGF) is the most vital member of the fibroblast growth factor (FGF) family, and its receptor is a tyrosine kinase. bFGF can stimulate the proliferation and migration of endothelial cells, smooth muscle cells, and fibroblasts; enhance the synthesis of extracellular matrix (ECM) components such as collagen; and induce cell migration, angiogenesis, and granulation tissue formation (24). IL-6 is an inflammatory cytokine synthesized and secreted by various cells, playing a crucial role in the immune regulation of the body, and is a key inflammatory neurotransmitter and regulatory factor in the body (25). TNF-*α* is a multifunctional Th1 cytokine, primarily produced by activated mononuclear macrophages. When the body is injured and stimulated, TNF-α is rapidly released, stimulating host defense mechanisms and chemotactic immune cells to clear trauma infections (26). MMPs are zinc ion-dependent proteolytic enzymes. Research has shown that MMP-9 can facilitate fibroblast activation and collagen fiber proliferation while degrading collagen and elastin, indirectly promoting extracellular matrix (ECM) synthesis (27). Tissue inhibitors of metalloproteinases (TIMPs) have various physiological, biochemical, and biological functions, including inhibition of MMP activation, enhancement of cell growth and matrix binding, suppression of angiogenesis, and induction of cell apoptosis (28). TIMPs, as crucial regulatory cytokines of MMPs, closely bind to them and jointly maintain the stability of the ECM and internal environment through their balance (29). Inherently, the TGF-β1/Smad3-VEGF signaling pathway plays an important role in wound healing and angiogenesis (30).

**Figure 4:**
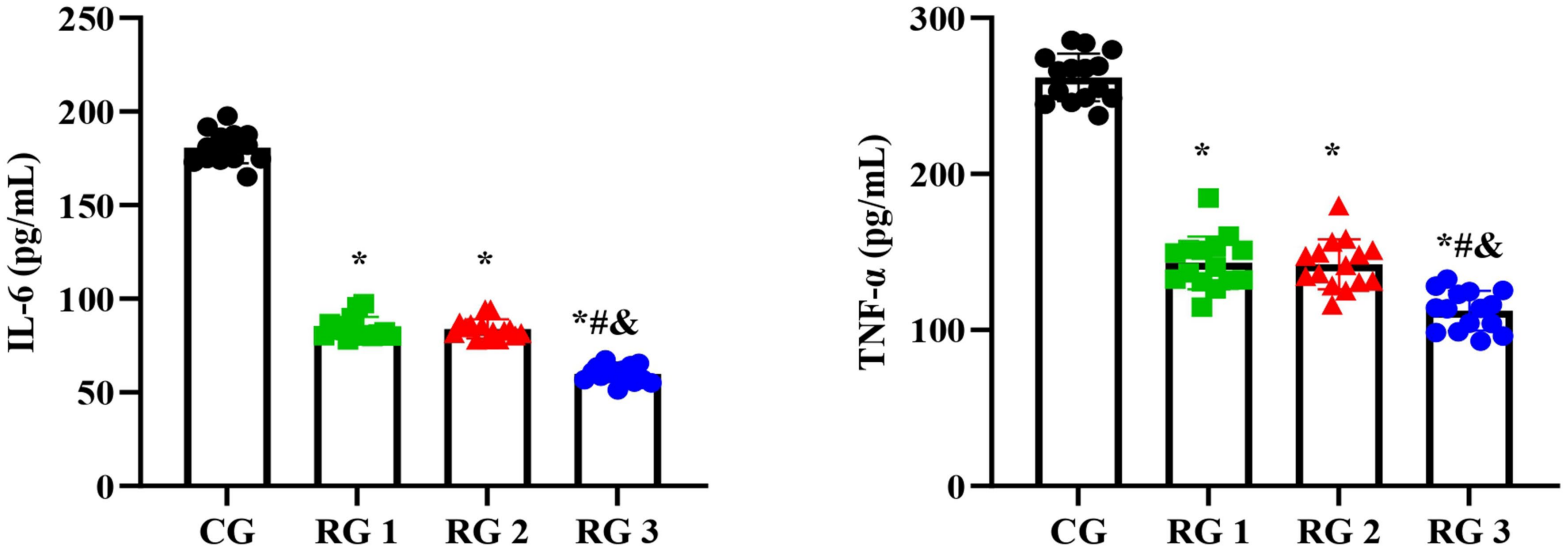
bFGF levels in the four groups. Versus the CG, *p < 0.05; versus RG 1, #*p* < 0.05; versus RG 2, &*p* < 0.05.

**Figure 5:**
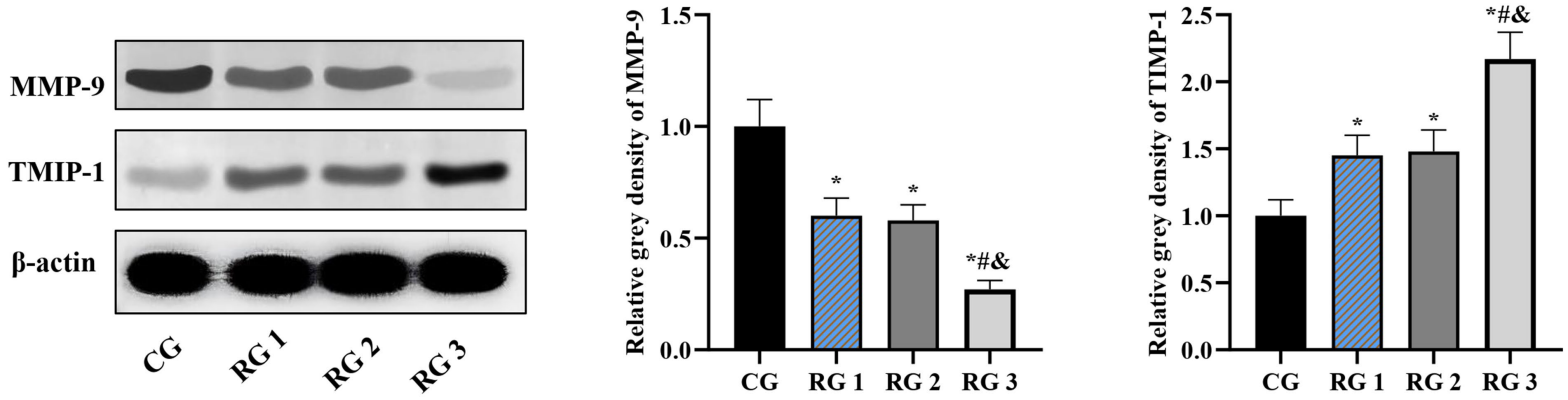
Serum IL-6 and TNF-*α* levels in the four groups. Versus the CG, *p < 0.05; versus RG 1, #*p* < 0.05; versus RG 2, &*p* < 0.05.

**Figure 6:**
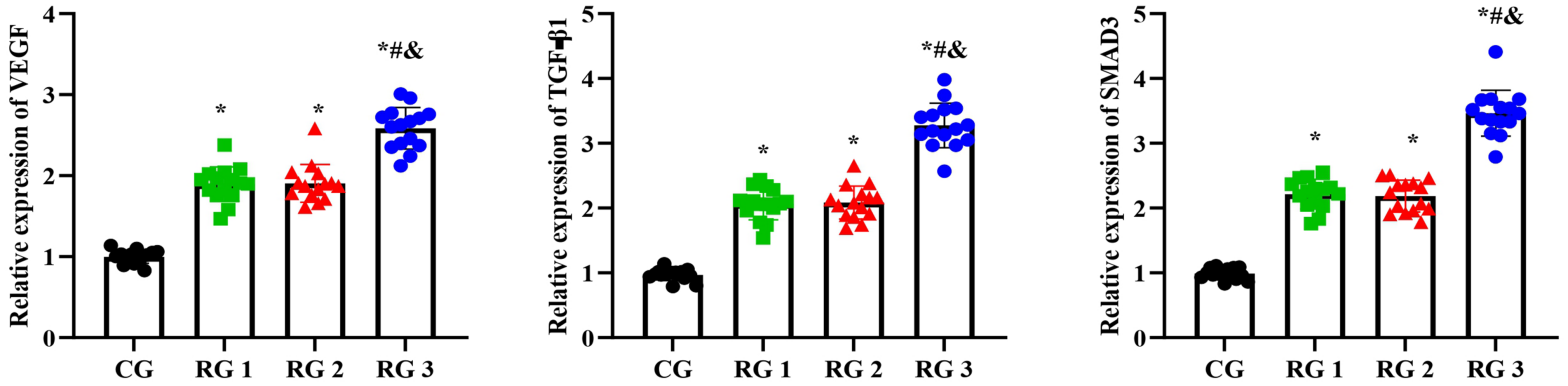
MMP-9 and TIMP-1 protein levels in the four groups. Versus the CG, *p < 0.05; versus RG 1, #*p* < 0.05; versus RG 2, &*p* < 0.05.

**Figure 7:**
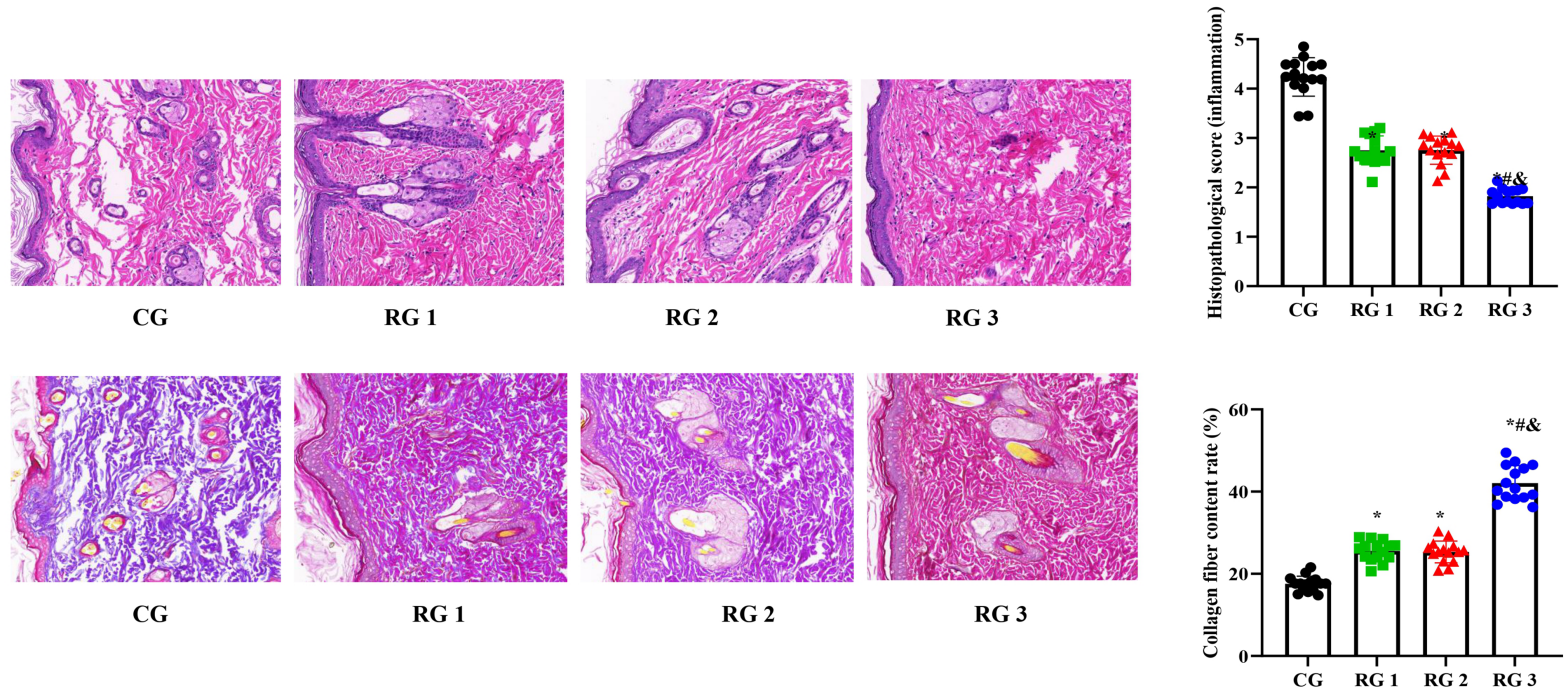
VEGF, TGF-β1, and SMAD3 mRNA levels in the four groups. Versus the CG, *p < 0.05; versus RG 1, #*p* < 0.05; versus RG 2, &*p* < 0.05.

In this study, the results suggested that on day 14 of treatment, tissue bFGF levels in all research groups were higher than those in the CG (*p* < 0.05). The tissue bFGF level in RG 3 was higher than that in RG 1 and RG 2 (*p* < 0.05; Figure 4; Tables 5, 6). At the same time, on day 14 of treatment, serum IL-6 and TNF-*α* levels in all research groups were lower than those in the CG (*p* < 0.05). Serum IL-6 and TNF-α levels in RG 3 were lower than those in RG 1 and RG 2 (*p* < 0.05; Figure 5; Table 7). In addition, on day 14 of treatment, tissue MMP-9 levels in both research groups were lower than those in the CG, and tissue TIMP-1 levels in both research groups were higher than those in the CG (*p* < 0.05). The tissue MMP-9 level in RG 3 was lower than that in RG 1 and RG 2, and the tissue TIMP-1 level in RG 3 was higher than that in RG 1 and RG 2 (*p* < 0.05; Figure 6; Table 8). Moreover, on day 14 of treatment, compared to the CG, the mRNA levels of VEGF, TGF-β1, and SMAD3 were higher in all research groups (*p* < 0.05), and the levels in RG 3 were higher than those in RG 1 and RG 2 (*p* < 0.05; Figure 7; Table 9). These results indicate that the combination of collagen sponge and SP can enhance maxillofacial trauma healing in rats by promoting angiogenesis and granulation tissue formation, suppressing inflammatory responses, and modulating ECM stability. This effect may be related to the TGF-β1/Smad3-VEGF signaling pathway.

**Table 5:** Positive rate of bFGF in the four groups (%).

Groups	*n*	Positive rate of bFGF
CG	15	0.28 ± 0.02
RG 1	15	0.33 ± 0.06^*^
RG 2	15	0.31 ± 0.05^*^
RG 3	15	0.46 ± 0.10^&#*^

Versus the CG, *p < 0.05; versus RG 1, #*p* < 0.05; versus RG 2, &*p* < 0.05.

**Table 6:** Relative gray density of bFGF.

Groups	*n*	bFGF
CG	15	1.00 ± 0.12
RG 1	15	1.72 ± 0.17^*^
RG 2	15	1.75 ± 0.18^*^
RG 3	15	2.85 ± 0.30^&#*^

Versus the CG, *p < 0.05; versus RG 1, #*p* < 0.05; versus RG 2, &*p* < 0.05.

**Table 7:** Serum IL-6 and TNF-α levels in the four groups (pg/mL).

Groups	*n*	IL-6	TNF-α
CG	15	180.74 ± 8.44	261.83 ± 15.30
RG 1	15	84.38 ± 5.84^*^	143.07 ± 16.91^*^
RG 2	15	83.95 ± 5.09^*^	142.20 ± 15.98^*^
RG 3	15	59.97 ± 4.35^&#*^	112.38 ± 12.71^&#*^

Versus the CG, *p < 0.05; versus RG 1, #*p* < 0.05; versus RG 2, &*p* < 0.05.

**Table 8:** Relative gray density of MMP-9 and TMIP-1.

Groups	*n*	MMP-9	TMIP-1
CG	15	1.00 ± 0.12	1.00 ± 0.12
RG 1	15	0.60 ± 0.08^*^	1.45 ± 0.15^*^
RG 2	15	0.58 ± 0.07^*^	1.48 ± 0.16^*^
RG 3	15	0.27 ± 0.04^&#*^	2.17 ± 0.20^&#*^

Versus the CG, *p < 0.05; versus RG 1, #*p* < 0.05; versus RG 2, &*p* < 0.05.

**Table 9:** Relative mRNA levels of VEGF, TGF-β1, and SMAD3 in the four groups.

Groups	*n*	VEGF	TGF-β1	SMAD3
CG	15	0.99 ± 0.08	0.97 ± 0.08	0.99 ± 0.08
RG 1	15	1.89 ± 0.21^*^	2.06 ± 0.24^*^	2.21 ± 0.22^*^
RG 2	15	1.90 ± 0.23^*^	2.08 ± 0.25^*^	2.18 ± 0.24^*^
RG 3	15	2.58 ± 0.25^&#*^	3.27 ± 0.34^&#*^	3.46 ± 0.35^&#*^

Versus the CG, *p < 0.05; versus RG 1, #*p* < 0.05; versus RG 2, &*p* < 0.05.

In conclusion, collagen sponge and SP exert unparalleled advantages in enhancing trauma healing and mitigating scar tissue proliferation compared to other materials. In this study, collagen sponge was combined with SP for the first time, markedly accelerating maxillofacial trauma repair and reducing scar formation after trauma repair. This effect may be related to the TGF-β1/Smad3-VEGF signaling pathway.

## Data Availability

The datasets presented in this study can be found in online repositories. The names of the repository/repositories and accession number(s) can be found in the article/supplementary material.
